# *N ^6^*-methyladenosine demethylase ALKBH5: a novel regulator of proliferation and differentiation of chicken preadipocytes: ALKBH5 regulated preadipocyte proliferation and differentiation

**DOI:** 10.3724/abbs.2021007

**Published:** 2021-12-23

**Authors:** Qi Zhang, Bohan Cheng, Haixu Jiang, Huili Zhang, Hui Li

**Affiliations:** 1.Key Laboratory of Chicken Genetics and Breeding Ministry of Agriculture and Rural Affairs Harbin 150030 China; 2.Key Laboratory of Animal Genetics Breeding and Reproduction Education Department of Heilongjiang Province Harbin 150030 China; 3.College of Animal Science and Technology Northeast Agricultural University Harbin 150030 China

**Keywords:** ALKBH5, chicken, preadipocyte, proliferation, differentiation

## Abstract

Previous studies have reported that the *N ^6^*-methyladenosine demethylase ALKBH5 can regulate adipogenesis in humans. However, its function in birds remains unclear. In this study we aimed to explore the expression and function of the *ALKBH5*gene in chicken adipose tissue. The results showed that *ALKBH5* is widely expressed in various chicken tissues, and the expression of *ALKBH5* is relatively higher in abdominal adipose tissue. In addition, the expression of *ALKBH5* in abdominal adipose tissue of lean broilers is higher than that in fat broilers at 2 and 3 weeks of age. Moreover, the proliferation and differentiation of preadipocytes are associated with reduced and increased expression of *ALKBH5*, respectively. We also found that *ALKBH5*knockout promotes preadipocyte proliferation, as evidenced by an increase in cell viability, DNA replication activity, G _1_-S cell cycle progression, and the expressions of *Ki67* and *PCNA*. Furthermore, after knockout of *ALKBH5*, the lipid droplet accumulation and the expression of *PPARγ*, *A-FABP*, and *FAS* are reduced significantly. Thus, our results indicated that ALKBH5 is a novel regulator of proliferation and differentiation of chicken preadipocytes.

## Introduction

The prevalence of obesity and the associated metabolic diseases have been rising over the past several decades globally [1]. Obesity has become a significant public health problem that develops heart disease, type 2 diabetes, and cancer in humans [ 2, 3]. Obesity not only is harmful to human health but also has adverse effects on animal production performance. With the rapid growth of broiler chickens, abdominal fat deposition increases, resulting in many adverse consequences, including decreased feed efficiency, reproductive performance, and meat quality [ 4– 6].

Thus, it is vital to understand the molecular and genetic basis of adipose tissue growth and development to solve the issue of excessive fat deposition. The expansion of the adipose tissue mass is caused by an increase in the number and size of adipocytes. The adipocyte number is regulated by the commitment of mesenchymal stem cells (MSCs) to the adipocyte lineage as well as by the preadipocyte proliferation. In contrast, the size of the adipocytes is regulated by preadipocyte differentiation [ 7, 8]. Over the past few decades, the regulatory mechanisms of adipose tissue development and fat deposition, such as transcription factors, DNA methylation, and histone modification, have been extensively studied, and a series of essential progresses have been made [ 9– 11]. In addition to the chemical modification of DNA and proteins, RNA modification has become a research hotspot in the field of epigenetics in recent years. So far, more than 100 types of chemical modifications of RNA have been identified, with *N ^6^*-methyladenosine (m ^6^A) methylation being the most pervasive modification in eukaryotes [12]. In mammals, emerging evidence shows that m ^6^A modification plays a critical role in fat deposition and hepatic lipid metabolism [ 13– 15]. However, there are many differences in lipogenesis pattern between birds and mammals [ 16– 18]. To date, the function of m ^6^A modification in adipose deposition in birds is still largely unknown.

The dynamic and reversible regulation of m ^6^A is coordinated by multiple “writers” and “erasers”. It is catalyzed by a large RNA methyltransferase complex containing the methyltransferase-like (METTL) enzymes METTL3, METTL14, and Wilms tumor 1-associated protein (WTAP) [ 19, 20] and removed by two demethylases, i.e., fat mass and obesity-associated protein (FTO) and α-ketoglutarate dependent dioxygenase AlkB homolog 5 (ALKBH5) [ 21, 22]. Recently, a study has reported that ALKBH5 regulates adipogenic differentiation of human MSCs [23]. However, its function in birds is unclear.

In this study, we investigated the expression pattern and function of the *ALKBH5* gene in chicken adipose tissue by characterizing the tissue expression of *ALKBH5*, analyzing the difference in its expression in abdominal fat tissue between fat and lean chickens, and exploring its roles in the proliferation and differentiation of preadipocytes.

## Materials and Methods

### Experimental birds and management

Animal studies were conducted following the Guidelines for the Care and Use of Experimental Animals established by the Ministry of Science and Technology of the People’s Republic of China (#2006-398) and were approved by the Laboratory Animal Management Committee and the Institutional Biosafety Committee of Northeast Agricultural University (Harbin, China). Fifty six male birds (lean line, *n*=28, and fat line, *n*=28) from the 19 ^th^ generation (G _19_) and 4 male birds (fat line) from the 23 ^rd^ generation (G _23_) of the Northeast Agricultural University High and Low Fat (NEAUHLF) lines were used. NEAUHLF has been chosen since 1996 using plasma very-low-density lipoprotein concentration and abdominal fat percentage (AFP) as the selection criteria: AFP (%)=AFW/BW7×100%, where AFW is abdominal fat weight, and BW7 is body weight at 7 weeks of age. The breeding procedure was described in a previous study [24]. All birds used in this study were kept under similar environmental conditions and had free access to food and water. All birds received the starter feed [metabolizable energy (ME): 3000 kcal/kg; crude protein (CP): 210 g/kg] from hatching to 3 weeks of age, and fed with a grower diet (ME: 3100 kcal/kg; CP: 190 g/kg) from 4 to 7 weeks of age.

### Tissue collection

In total, 56 male birds (4 birds for each broiler line, aged 1–7 weeks) from G _19_ and four male birds (all for the fat broiler line, aged 7 weeks) from G _23_ were sacrificed after fasting for 10 h, and the AFP was calculated in G _19_ (Supplementary Figure S1). At the end of each week, the abdominal fat tissue was collected. For birds sacrificed at 7 weeks of age from G _23_, 19 other tissue samples, including pectoralis muscle (PM), leg muscle (LM), liver (L), testis (Te), heart (H), pancreas (Pa), duodenum (D), gizzard (G), Cecum (C), cerebrum(Cr), kidney (K), subcutaneous fat (SF), crop fat (CF), abdominal fat (AF), gizzard fat (GF), spleen (Sp), proventriculus (P), jejunum (J), ileum (I) and mesentery fat (MF) were also collected. After wash with 0.75% NaCl, all the tissues were collected, snap-frozen in liquid nitrogen, and stored at –80°C until further use.

### Cell culture and differentiation induction

Chicken stromal-vascular cells (SV) and fat cells (FC) were isolated according to the following procedure. First, abdominal fat tissue (3–5 g) was isolated from 12-day-old chickens, minced, and incubated with 2 mg/mL collagenase I (Sigma-Aldrich, St Louis, USA) with shaking for 1 h at 37°C. The suspension was then passed through a 100-μm and a 600-μm nylon cell strainer (BD Falcon, New York, USA), respectively, to remove undigested tissue. The filtrate was centrifuged at 200 *g* for 10 min at room temperature. The top layer (fat cell fraction) and the pellet (stromal-vascular cell fraction) were collected as chicken mature adipocytes and preadipocytes, respectively.

Chicken ICP2 preadipocyte cell line was preserved in our laboratory [25]. The ICP2 and ALKBH5-KO cells were plated at 1× 10 ^4^ cells/cm ^2^ in DMEM/F12 medium (Gibco, New York, USA) containing 10% FBS (Biological Industries, Kibbutz Beit Haemek, Israel) and 1% penicillin-streptomycin (Gibco). The cells were grown in a standard humidified incubator at 37°C with 5% CO _2_. Once the cells achieved > 90% confluency, they were passaged and plated at 6×10 ^4^ cells/well in 6-well plates. When the plated cells reached 50% confluency, differentiation was induced by addition of fresh differentiation medium (DMEM/F12 medium containing 10% FBS and 160 μM oleate).

### Genome editing

Three gRNAs were designed in the exon 2 of the chicken *ALKBH5* gene by using online software CRISPOR ( http://crispor.tefor.net/). The sequences of gRNAs are as follows: ALKBH5-g1, 5′-ACCGCCGCTTACGCTCGTAGGGG-3′; ALKBH5-g2, 5′-CGAAGCTCGCATTGACGATGTGG-3′; and ALKBH5-g3, 5′-GTGATCAACGACTATCAGCCCGG-3′. Then the gRNAs were ligated into the Cas9 expression vector using Cas9/gRNA construction kit for poultry (Viewsolid, Beijing, China), respectively. The constructed Cas9/gRNA plasmid could simultaneously express Cas9 protein through pCAG promoter and gRNA through poultry U6 promoter, with GFP and zeocin screening markers. The three plasmids, Cas9/ALKBH5-g1, Cas9/ALKBH5-g2, and Cas9/ALKBH5-g3, were transfected into the ICP2 cells respectively using Lipofectamine 3000 (Invitrogen, Carlsbad, USA) according to manufacturer’s instructions. Next, 24 h after transfection, cells were screened by 200 μg/mL zeocin for 5 days. Then the cells were collected for DNA extraction. The target sites of gRNA were amplified by PCR, and the PCR products were digested by T7E1 (Vazyme, Nanjing, China) to identify the cleavage efficiency of each Cas9/gRNA plasmid. The PCR primers used were ALKBH5-text forward and reverse, and the primer sequences are listed in Table 1. The Cas9/gRNA plasmid with the highest cleavage efficiency was chosen for positive cell screening based on the presence of the *GFP* gene in the construct. Briefly, 48 h after transfection with the selected Cas9/gRNA plasmid, GFP-positive cells were sorted into 96-well plates (one cell/well) by flow cytometry (FACSARIA; BD Biosciences, San Jose, USA). The types of ALKBH5 gene editing in the monoclonal cell line were detected by TA cloning and sequencing. The knockout efficiency of *ALKBH5* was evaluated by western blot analysis.

**Table:** **Table1** Sequences of primers used in this study

Gene	Accession No.	Primer sequence (5 *′*→3 *′*)
*ALKBH5-text*	NM_001257201	F: GTGCTTGCCCTCACGTTGTC
		R: TGGGCCGCTCGAAGATATG
*ALKBH5*	NM_001257201	F: GCGCTCAGTCCTCTTACCAA
		R: ATTCCTCAGTGTCGCCTCATT
*FAS*	NM_205155	F: AAGGCGGAAGTCAACGG
		R: TTGATGGTGAGGAGTCG
*A-FABP*	NM_204290	F: ATGTGCGACCAGTTTGT
		R: TCACCATTGATGCTGATAG
*PPARγ*	NM_001001460	F: GTGCAATCAAAATGGAGCC
		R: CTTACAACCTTCACATGCAT
*PCNA*	NM_204170	F: GTGCTGGGACCTGGGTT
		R: CGTATCCGCATTGTCTTCT
*Ki67*	NM_205505	F: AGGTCCGTTCCCTCGTT
		R: CATTGTCGTCTGGGTCATC
*TBP*	NM_205103	F: GCGTTTTGCTGCTGTTATTATGAG
		R: TCCTTGCTGCCAGTCTGGAC

F, forward; R, reverse.

### Lipid staining and measurement of lipid droplet accumulation

Lipid droplets were stained with Oil red O (Sigma, Burlington, USA). First, the cells were washed thrice with PBS and then fixed with 4% paraformaldehyde for 30 min. After fixation, 4% paraformaldehyde was discarded and cells were washed thrice with PBS, and then stained with Oil red O working solution (Oil red O stock solution:distilled water=3:2) at room temperature for 15 min, followed by washing three times with PBS. Finally, the cells were washed with 60% isopropanol for 10–20 s, thrice with distilled water, observed microscopically using an inverted fluorescence microscope (Leica, LEICA DMIRB), and images were captured.

Lipid droplet accumulation was measured by Oil red O extraction assay [26]. The Oil red O in the stained cells was dissolved using 100% isopropanol for 15 min and then the absorbance was measured at 510 nm. Before lipid staining, the cell count in different groups was assessed based on absorbance at 490 nm by the cell proliferation assay using the CellTiter 96® AQueous One Solution (Promega, Madison, USA), and the cell count was used to normalize the extraction data. The lipid droplet accumulation was presented as a ratio of OD _510_/OD _490_.

### RNA extraction and reverse transcription-quantitative polymerase chain reaction (qRT-PCR)

Total RNA of tissues (100 mg each) and cells were extracted using a Trizol reagent kit (Invitrogen, Carlsbad, USA). Reverse transcription was performed using 1 μg of total RNA from each sample and the PrimeScript™ RT reagent kit with gDNA Eraser (Perfect Real Time; Takara, Dalian, China), and a 7500 Real-time PCR System (Applied Biosystems, Foster City, USA) was used to conduct qRT-PCR. FastStart Universal SYBR Green Master Mix (Roche, Indianapolis, USA) was used for qPCR using 1 μL of cDNA and appropriate volumes of specific primers, in a final 10 μL volume. The qPCR cycling conditions were as follows: 95°C for 10 min; 95°C for 15 s, 60°C for 1 min, 40 cycles. Triplicate reactions were performed to ensure accuracy. Gene expression was normalized to that of TATA-box binding protein (TBP), and the 2 ^−ΔCT^ method was used for expression calculations [27], ΔCT=CT _target gene_–CT _TBP_. The primer sequences are shown in Table 1.

### Western blot analysis

Total protein was extracted from the cells by using RIPA buffer (Beyotime, Shanghai, China) supplemented with protease inhibitor (Beyotime). The total protein was added into a 6× denaturing loading buffer, boiled for 5 min, separated by 12% SDS-PAGE, and transferred to an Immun-Blot PVDF membrane (Millipore, Billerica, USA). After incubation with a primary antibody against chicken PCNA (1:1000; Abcam, Cambridge, USA) or chicken β-actin (1:1000; Beyotime), the membranes were washed and incubated with a horseradish peroxide-conjugated secondary antibody (1:5000; Beyotime). Specific protein bands were visualized using the ECL detection kit (HaiGene, Harbin, China) in a chemiluminescence system (Sagecreation), and band intensity was quantified with the ImageJ software (NIH, Bethesda, USA).

### m ^6^A dot blot assay

Total RNA (200 ng) from the cells treated with DNase I (Sigma, Burlington, USA) was spotted to a nylon membrane (Thermo Fisher, Waltham, USA), followed by UV crosslinking at UV 254 nm, 1200 J, for 1 min at room temperature. After being blocked in PBS with 1% Tween-20 (PBST) containing 5% non-fat milk for 4 h at room temperature, the membrane was incubated with a specific anti-m ^6^A antibody (1:2000; Synaptic Systems, Göttingen, Germany) overnight at 4°C. Then the horseradish peroxide-conjugated secondary antibody was added to the blots and incubated for 1 h at room temperature. The membrane was developed using an ECL detection kit (HaiGene) and scanned in a chemiluminescence system. The relative signal density of each dot was quantified with the ImageJ software.

### Cell proliferation assay

CellTiter 96® AQueous One Solution Cell Proliferation Assay kit (Promega) and EdU cell proliferation detection kit (Ribobio, Hangzhou, China) were used to detect cell proliferation. Cell suspension (2 mL) was inoculated into a 6-well plate, and the plate was cultured in an incubator for 12 h. Next, 400 μL of test reagent was added to each well. The plate was incubated at 37°C for 1 h. The absorbance of each sample was measured at a wavelength of 490 nm and detected using a microplate reader (Molecular Devices, Sunnyvale, USA). Cell proliferation was also assessed using the EdU cell proliferation detection kit according to the manufacturer’s instructions.

### Cell cycle assay

The cells were digested with 200 μL of trypsin (Gibco, New York, USA). and collected into a centrifuge tube. After centrifugation at 800 *g* for 5 min, the cells were precipitated. Next, the cells were resuspended in 1 mL of PBS. After centrifugation at 800 *g* for 5 min, the supernatant was discarded, and the cells were resuspended in 3 mL of PBS, and then 7 mL of precooled absolute ethanol was added for immobilization. The cells were fixed in 70% absolute ethanol at 4°C for 24 h. After centrifugation at 800 *g* for 5 min, the supernatant was discarded, and the fixed cells were suspended in 1 mL of PBS. After centrifugation at 800 *g* for 5 min, the supernatant was discarded, and 0.5 mL of propidium iodide staining solution was added to each tube, and the cells were incubated at 37°C in the dark for 30 min. Then the cells were stored at 4°C or ice, and subject to flow cytometric analysis on the flow cytometry.

### Statistical analysis

All experiments were repeated thrice. All data are shown as the mean±SD. Student’s *t*-test was used to compare results between two groups. When more than two groups were compared, a generalized linear model (GLM) procedure followed by Turkey’s HSD test was used according to the model: *Y*= *μ*+ *F*+ *e*, in which *Y* is the dependent variable ( *ALKBH5* mRNA expression level), *μ* is the population mean, *F* is various factors (time point of proliferation or differentiation of preadipocyte or broiler age) as the fixed effect, and *e* is the random residual effect. JMP v11.0 (SAS Institute, Inc., Cary, USA) was used for all analyses, and the threshold of significance was set at *P<*0.05.

## Results

### Characterization of the tissue expression of *ALKBH5*

**Figure 1:**
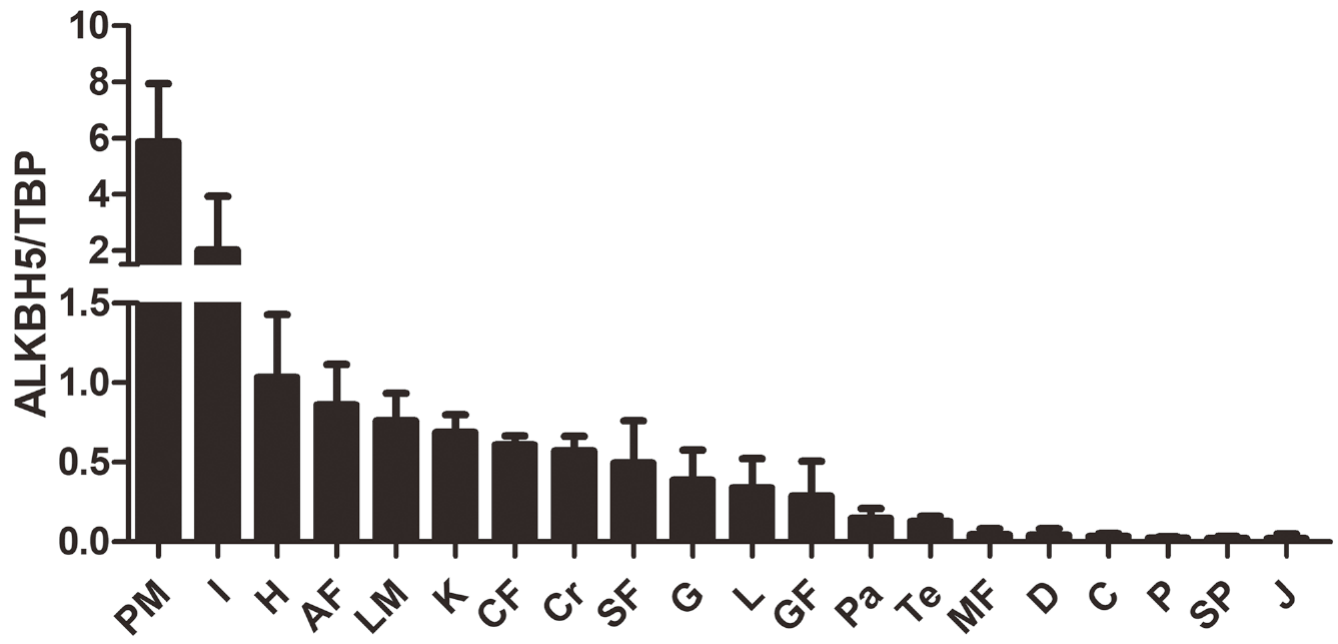
Figure1 Tissue expression characterization of chicken ALKBH5 in 7-week-old fat male broilers of G _23_ PM, pectoralis muscle; LM, leg muscle; L, liver; Te, testis; H, heart; Pa, pancreas; D, duodenum; G, gizzard; C, Cecum; Cr, cerebrum; K, kidney; SF, subcutaneous fat; CF, crop fat; AF, abdominal fat; GF, gizzard fat; Sp, spleen; P, proventriculus; J, jejunum; I, ileum; and MF, mesentery fat.

In order to investigate whether *ALKBH5* gene is highly expressed in chicken abdominal adipose tissue, we constructed an ALKBH5 expression profile. qRT-PCR results showed that *ALKBH5* mRNA was expressed in all of the tissues tested from 7-week-old fat male birds of G _23_ ( Figure 1). The expression of *ALKBH5* transcript was relatively high in the pectoralis muscle, ileum, heart, abdominal fat, leg muscle, kidney, crop fat, cerebrum, and subcutaneous fat; medium in the gizzard, liver, and gizzard fat; and low in the pancreas, testis, mesentery fat, duodenum, cecum, proventriculus, spleen, and jejunum ( Figure 1).

### Expression pattern of the *ALKBH5* during adipose tissue development of fat and lean broilers

**Figure 2:**
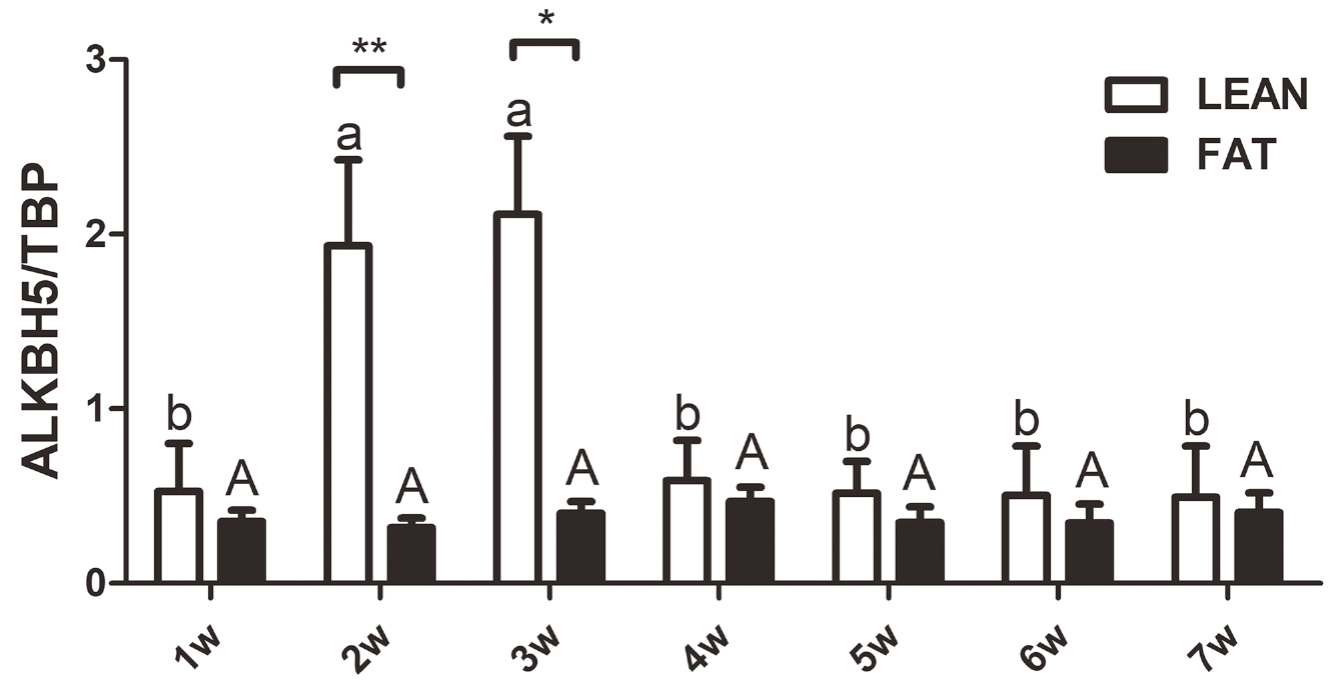
Figure2 Expression pattern of ALKBH5 during chicken abdominal adipose development “a” and “b”, the different lowercase letters above columns indicate significant differences among various ages in the lean line (**P<0.05). “A”, the uppercase letter above columns indicates no significant difference among various ages in the fat line (P>0.05).

We further analyzed the *ALKBH5*expression in abdominal fat tissues of 1- to 7-week-old broilers from G _19_ by qRT-PCR, and the results showed that *ALKBH5*was expressed in all the chicken abdominal fat tissues tested. In addition, in the lean line, the expression of *ALKBH5*was increased during the early stages of development (1 to 3 weeks of age), decreased at 4 weeks of age, and then maintained a stable expression ( Figure 2). Meanwhile, the expression of *ALKBH5* was maintained at a stable medium level during the development of adipose tissue in the fat line ( Figure 2). Moreover, a comparison of *ALKBH5* expression in the abdominal fat tissue between fat and lean broilers at each age showed that, at 2 and 3 weeks of age, the expression of *ALKBH5*was significantly higher in the lean chickens than in the fat chickens ( *P<*0.05; Figure 2). These data indicated that the expression of *ALKBH5* is associated with fat deposition at the early stage of chicken abdominal adipose tissue development.

### Establishment of chicken *ALKBH5*-knockout preadipocyte cell line

**Figure 3:**
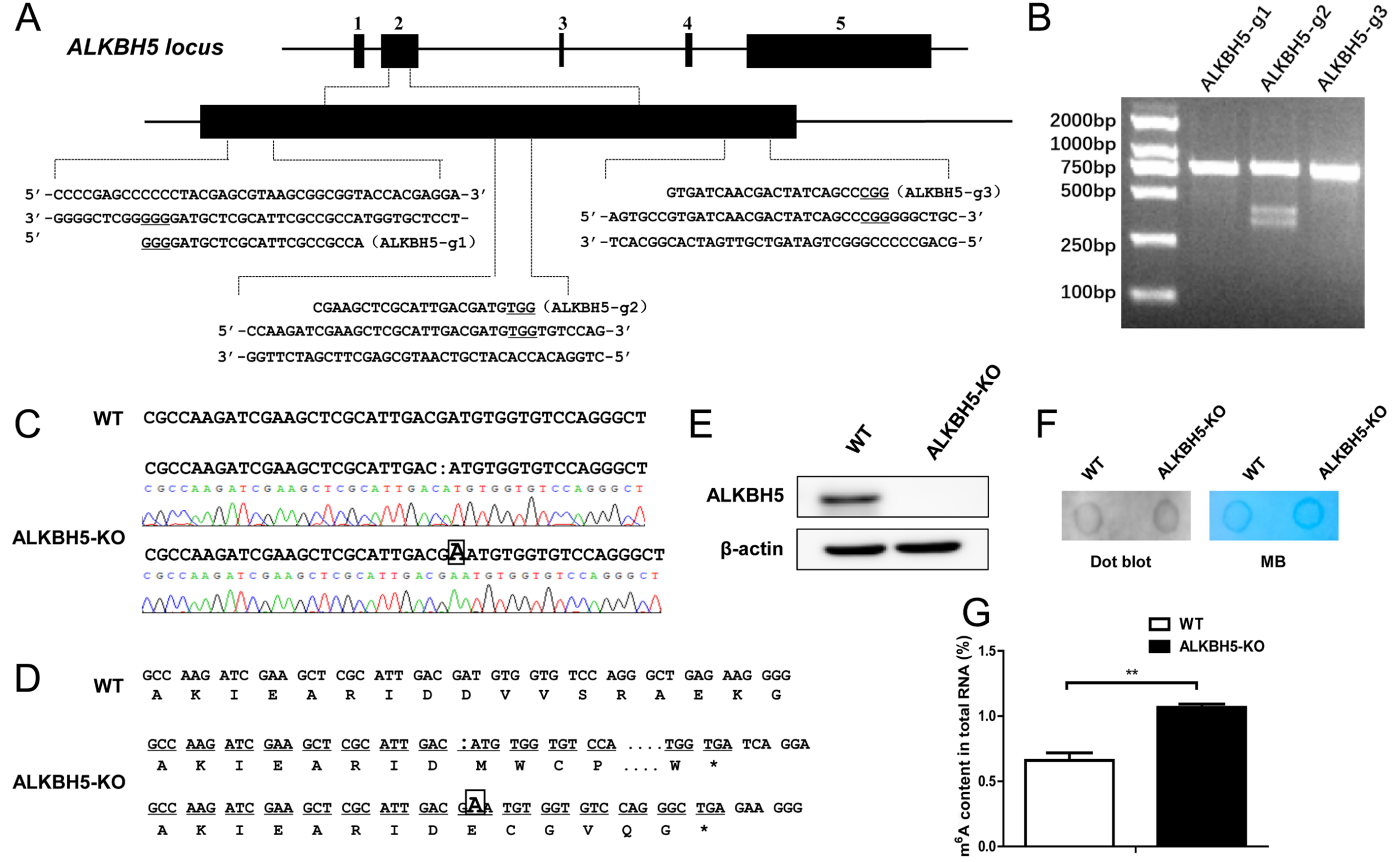
Figure3 Establishment of *ALKBH5*-knockout preadipocyte cell line (A) Design of the gRNAs. The black box represents the exon, and the underline represents the PAM sequence. (B) The cleavage efficiencies of the gRNAs were detected using the T7E1 enzyme. (C) The detection of genome editing types by Sanger sequencing. (D) Open reading frame analysis of ALKBH5 gene in ALKBH5-knockout ICP2 cells. A colon represents the deletion of a base, a box represents the insertion of a base, and a star represents the stop codon. (E) The protein level of ALKBH5 was detected by western blot analysis. (F) The m6A content of total RNA in ALKBH5-knockout ICP2 cells was detected by dot blot using m6A antibody. MB, methylene blue staining (as loading control). (G) The m6A methylation level was quantified by gray value analysis. **P<0.01.

Next, we established a chicken preadipocyte line with *ALKBH5* knockout using CRISPR/Cas9 gene-editing technology to investigate the function of ALKBH5 in the proliferation and differentiation of chicken preadipocytes. Three gRNAs were designed in the exon 2 of the chicken *ALKBH5* gene. Figure 3A shows the sequences of the gRNAs. The results of T7E1 digestion showed that Cas9/ALKBH5-g2 vector had cleavage activity ( Figure 3B). Therefore, Cas9/ALKBH5-g2-transfected cells were selected for monoclonal screening. A total of three cell lines were obtained (named as ALKBH5-g2-1, -2, and -3). The results of Sanger sequencing showed that two monoclonal cell lines were wild-type, and one monoclonal cell line was genetically edited with one base deletion of *ALKBH5* exon 2 in a homologous chromosome and one base insertion of *ALKBH5* exon 2 in another homologous chromosome ( Figure 3C). We named this cell line as ALKBH5-KO. The results of sequence analysis showed a shift in the open reading frame of the ALKBH5-KO cell line, leading to the early termination of translation ( Figure 3D). Then western blot analysis was used to detect whether *ALKBH5* is knocked out in the ALKBH5-KO cell line. The results showed that ALKBH5 protein could not be detected in ALKBH5-KO ICP2 cells ( Figure 3E). Considering that ALKBH5 is an m ^6^A demethylase, we tested whether *ALKBH5* knockout changes the global m ^6^A level. The results of m ^6^A dot blot showed that the global m ^6^A level in the *ALKBH5*-knockout preadipocytes was significantly higher than that in the wild-type preadipocytes ( *P<*0.01; Figure 3F,G). Thus, these results suggested that the chicken *ALKBH5-*knockout preadipocyte cell line was established successfully.

### Knockout of *ALKBH5* promotes chicken preadipocyte proliferation

**Figure 4:**
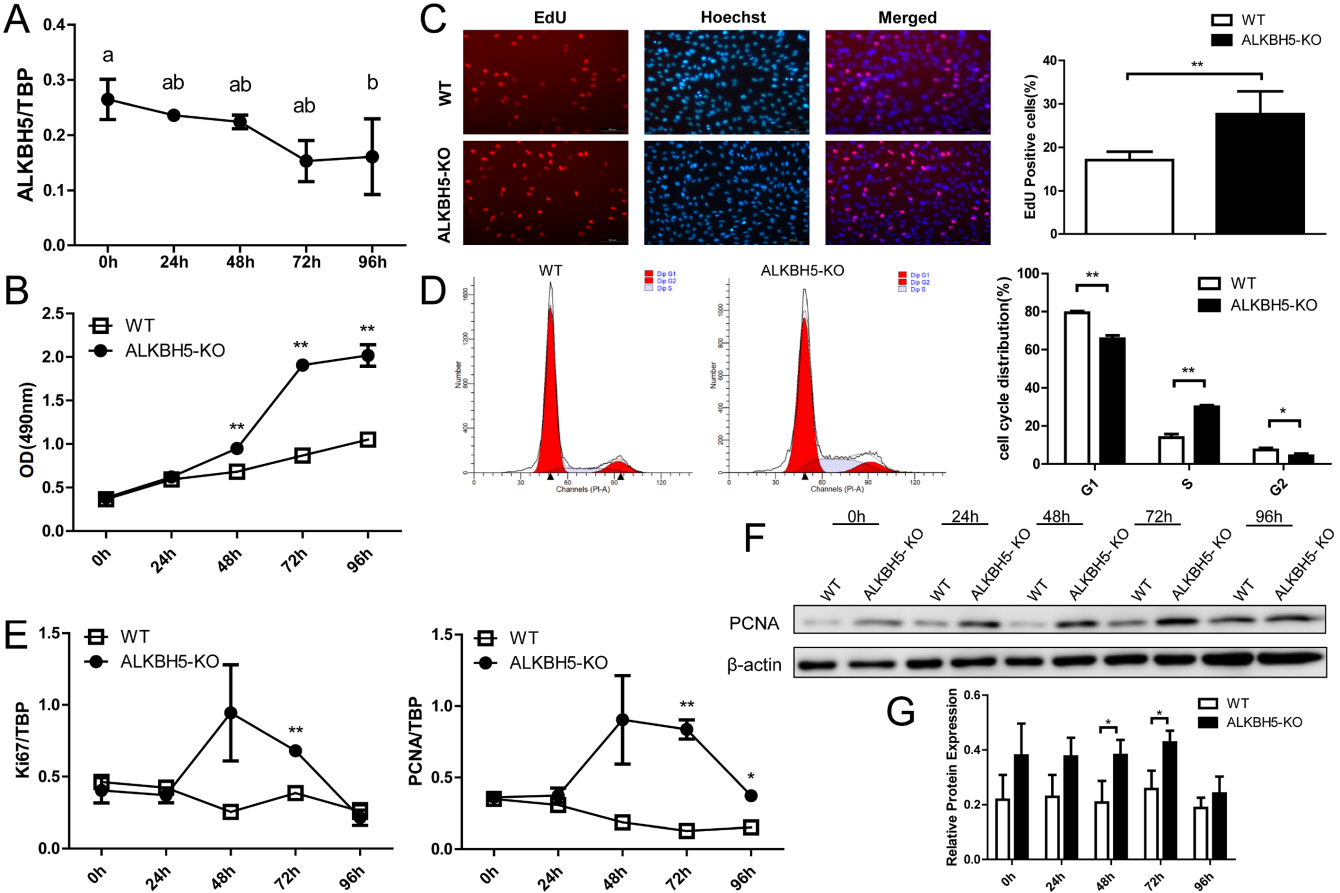
Figure4 Effects of *ALKBH5* knockout on ICP2 cell proliferation (A) The mRNA level of ALKBH5 during chicken preadipocyte proliferation was determined by qRT-PCR. Six hours after cell seeding was defined as 0 h of proliferation. "a" and "b", The different lowercase letters above columns indicate significant differences among various time points (Tukey’s HSD test, P<0.05). (B) Cell viability was analyzed. (C) DNA synthesis activity was analyzed using the EdU staining at 48 h of proliferation. EdU (red) was used to detect the proliferating cells by labeling the newly synthesized DNA, and Hoechst 33342 (blue) was used to measure the background by staining total cellular DNA. The ratio EdU/Hoechst was used to evaluate the cell proliferation rate. Scale bar: 100 μm. (D) The cell cycle was analyzed by flow cytometry at 48 h of proliferation. (E) The mRNA expression of Ki67 and PCNA was determined by qRT-PCR. (F) The protein expression of PCNA was detected by western blot analysis. (G) Quantitative analysis of PCNA protein expression levels. *P<0.05 and **P<0.01.

Next, the expression of *ALKBH5* was detected during the proliferation of ICP2 cells. The results of qRT-PCR showed that the expression of *ALKBH5* was decreased during the chicken preadipocyte proliferation ( *P<*0.05; Figure 4A), suggesting that ALKBH5 plays an inhibitory role in chicken preadipocyte proliferation. Thus, we performed the functional analysis of ALKBH5 using the wild-type ICP2 cells and the *ALKBH5*-knockout ICP2 cells to test this hypothesis. We analyzed the cell proliferation and the results showed that at 48, 72, and 96 h of the preadipocyte proliferation, the cell viability of *ALKBH5*-knockout ICP2 cells was significantly higher than that of the wild-type ICP2 cells ( *P<*0.01; Figure 4B). The results of EdU staining showed that the DNA replication activity of *ALKBH5*-knockout ICP2 cells was significantly higher than that of the wild-type ICP2 cells at 48 h of preadipocyte proliferation ( *P<*0.01; Figure 4C).

We further assayed the cell cycle by flow cytometry to investigate the role of ALKBH5 in chicken preadipocyte proliferation. The results showed that knockout of *ALKBH5* resulted in a significant decrease in the proportion of cells at G _1_ phase ( *P<*0.01), and an extremely significant increase in the proportion of cells in the S phase at 48 h of proliferation ( *P<*0.01; Figure 4D). Moreover, we detected the effect of *ALKBH5* knockout on the expression of proliferation markers Ki67 and PCNA. The results of qRT-PCR showed that the mRNA expression of *Ki67* in the *ALKBH5*-knockout ICP2 cells was significantly higher than that in the wild-type ICP2 cells at 72 h of proliferation ( *P<*0.01), and the mRNA expression of *PCNA* in the *ALKBH5-*knockout ICP2 cells was significantly higher than that in the wild-type ICP2 cells at 72 and 96 h of proliferation ( *P<*0.05; Figure 4E). Western blot analysis results showed that the expression of PCNA in the *ALKBH5*-knockout ICP2 cells was higher than that in the wild-type ICP2 cells at 48 and 72 h of proliferation ( *P*<0.05; Figure 4F,G). These results indicated that ALKBH5 inhibits chicken preadipocyte proliferation.

### Knockout of *ALKBH5* inhibits chicken preadipocyte differentiation

**Figure 5:**
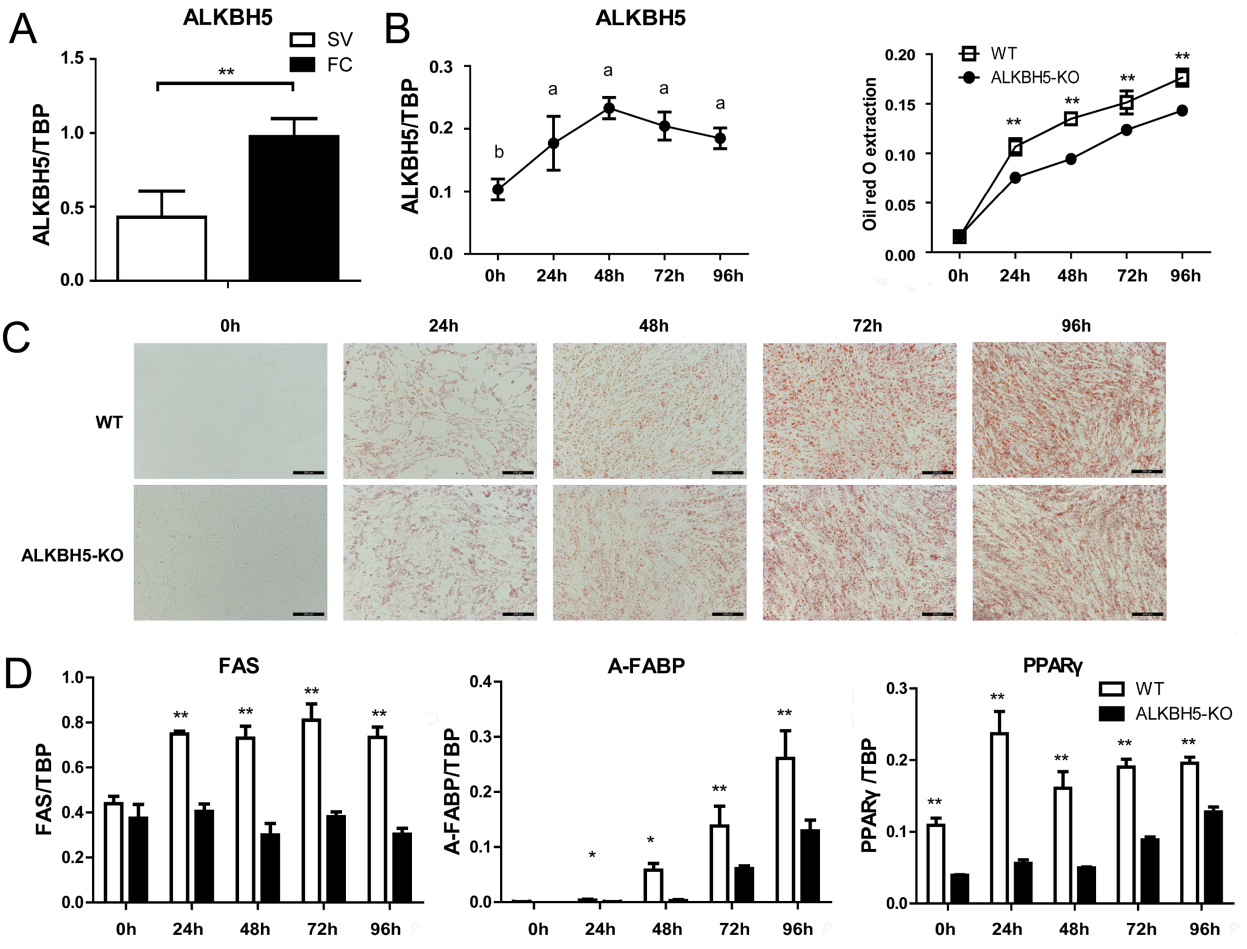
Figure5 Effects of *ALKBH5* knockout on the differentiation of ICP2 cells (A) Analysis of ALKBH5 mRNA level in chicken preadipocytes (SV) and mature adipocytes (FC). **P<0.01. (B) The mRNA level of ALKBH5 during chicken preadipocyte differentiation was determined by qRT-PCR. (C) Lipid droplet accumulation was measured by Oil red O staining and extraction assay. Scale bar: 200 μm. (D) The mRNA levels of PPARγ, A-FABP, and FAS were detected by qRT-PCR. *P<0.05 and **P<0.01. “a” and “b”, the different lowercase letters above columns indicate significant differences among various time points (Tukey’s HSD test, P<0.05).

To understand whether *ALKBH5* is involved in chicken preadipocyte differentiation, chicken preadipocytes (stromal-vascular cell fraction) and mature adipocytes (fat cell fraction) were isolated from the abdominal adipose tissues of broilers, and *ALKBH5* mRNA level was measured by qRT-PCR. *ALKBH5* was expressed in both chicken preadipocytes and mature adipocytes, and its expression was significantly higher in mature adipocytes than in preadipocytes ( *P*<0.01; Figure 5A). Then, the expression of *ALKBH5* was detected during the differentiation of ICP2 cells. The results of qRT-PCR showed that the expression of *ALKBH5* was increased during the chicken preadipocyte proliferation ( *P<*0.05; Figure 5B), suggesting that ALKBH5 plays a positive role in regulating chicken preadipocyte proliferation. Thus, we performed the functional analysis of ALKBH5 using the wild-type ICP2 cells and the *ALKBH5*-knockout ICP2 cells to test this hypothesis. We assessed both lipid accumulation and the expressions of pro-adipogenic differentiation genes, including peroxisome proliferator-activated receptor γ ( *PPARγ*), adipocyte fatty-acid binding protein ( *A-FABP*), and fatty acid synthase ( *FAS*) to investigate whether *ALKBH5* knockout affects the preadipocyte differentiation. Compared with the wild-type ICP2 cells, *ALKBH5*-knockout ICP2 cells exhibited a significant decrease in the intracellular lipid droplet accumulation at 24, 48, 72, and 96 h of preadipocyte differentiation, based on the results of oil red O staining and the quantitative assessment ( *P*<0.01; Figure 5C). Consistently, the mRNA expression of *PPARγ* was decreased at 0, 24, 48, 72, and 96 h of differentiation ( *P<*0.01; Figure 5D); the mRNA expressions of *A-FABP* and *FAS* were decreased at 24, 48, 72 and 96 h of differentiation ( *P<*0.05 or *P<*0.01; Figure 5D). These results indicated that ALKBH5 promotes chicken preadipocyte differentiation.

## Discussion

ALKBH5 is a member of the Alk B family and is a key enzyme that can remove RNA m ^6^A methylation [22]. ALKBH5 is mainly involved in spermatogenesis and carcinogenesis [ 22, 28, 29]. A previous study by Zheng *et al*. [22] showed that ALKBH5 was widely expressed in various mouse tissues, including the heart, brain, gonadal fat pads, liver, kidney, spleen, lung, and testis in mice. Similarly, the results of our study showed that the *ALKBH5*gene was widely expressed in chicken tissues. Notably, the expression of *ALKBH5* was relatively higher in abdominal fat than in other adipose tissues ( Figure 1), suggesting that ALKBH5 plays an important role in chicken abdominal fat deposition. Until now, there are no reports regarding the pattern of expression of ALKBH5 during adipose tissue development. Here, we determined the expression of ALKBH5 during adipose tissue development of fat and lean broilers to explore whether ALKBH5 is related to abdominal adipose development and fat deposition. Our results showed that the expression of ALKBH5 was dynamic during the growth and development of abdominal adipose tissue, and there was a significant difference in the expression of ALKBH5 between fat and lean chickens at 2 and 3 weeks of age ( Figure 2), suggesting that ALKBH5 is involved in chicken abdominal fat deposition, especially during the early stage of adipose development after birth. During the growth and development of abdominal adipose tissue, the expression pattern of ALKBH5 in fat and lean line broilers is not completely consistent ( Figure 2). This phenomenon is probably related to the difference in adipocyte development pattern in the abdominal adipose tissue between the two chicken lines [24].

Gene-editing technology is an important tool to study gene function. Although the early homologous recombination technology can effectively edit the target gene, the efficiency is very low. The emergence of artificial nuclease technology has improved the efficiency of genome editing. There are two kinds of artificial nuclease systems, ZFNs and TALENs, which have been previously used. However, ZFNs and TALENs are time-consuming and laborious in plasmid construction [ 30, 31]. In 2012, Cas9 was first proposed to cleave genomic DNA *in vitro* [32], and the construction of its plasmid vector is simpler and more efficient [33]. Subsequently, numerous studies emerged in 2013 to prove that the Cas9 system could effectively edit the genome in many cells and organisms. Currently, CRISPR/Cas9 system is mainly applied to human 293T cells, human pluripotent stem cells [34], zebrafish [35], mice [36], rats [37], pigs [38], rabbits [39], and frogs [40]. In addition, scientists have successfully edited the genome of chicken cells using the CRISPR/Cas9 system. Cheng *et al*. [41] successfully knocked out *TBK1* gene in chicken DF-1 cells using the CRISPR/Cas9 system. The *TBK1*-knockout cells exhibited normal morphology and maintained stable proliferation ability compared to wild-type cells. Zhang *et al*. [42] used the CRISPR/Cas9 system to knock down *Stra8* in DF-1 cells and chicken embryonic stem cells, which inhibited the embryonic stem cell differentiation into spermatogenic stem cells. Qin *et al*. [43] successfully knocked-in human epidermal growth factor (hEGF) on the chicken ovalbumin locus using the CRISPR/Cas9 system. The inserted hEGF cDNA could be expressed in primary oviduct cells, and the secreted hEGF promoted proliferation of HeLa cells [43]. Thus, we constructed a chicken *ALKBH5*-knockout preadipocyte line by using the CRISPR/Cas9 system to study the function of ALKBH5 in chicken preadipocyte proliferation and differentiation. The type of *ALKBH5*-editing of two homologous chromosomes in this cell line was the deletion of one base and insertion of one base, respectively, which caused frameshift mutation, leading to early termination of translation ( Figure 3C–E), and further confirmed that CRISPR/Cas9 gene-editing system could be used for chicken cells. In addition, consistent with our expectation, knockout of *ALKBH5* significantly increased the global m ^6^A level in ICP2 cells. This suggested that ALKBH5 acts as the m ^6^A demethylase in chickens, as in other species. However, further study using MeRIP-seq is needed to determine which transcripts have up-regulated m ^6^A methylation levels after knockout of *ALKBH5* in ICP2 cells.

In the present study, we found that the mRNA level of *ALKBH5*was decreased during the proliferation of ICP2 cells, suggesting that ALKBH5 is probably involved in chicken preadipocyte proliferation. Previous studies have demonstrated that ALKBH5 inhibits the proliferation of various cell types, such as human bladder cancer cells [44], human pancreatic cancer cells [45], and human hepatocellular carcinoma cells [46]. Our findings indicated that *ALKBH5* knockout enhanced chicken preadipocyte proliferation. Interestingly, our findings are different from some other reports showing that ALKBH5 promoted the proliferation of some cell types, including human glioblastoma stem-like cells [29], human renal cell carcinoma cells [47], and human lung adenocarcinoma cells [48]. This discrepancy suggested that ALKBH5 either promotes or inhibits cell proliferation, depending on the cell type.

Adipogenesis is a complex biological process regulated by genetic and epigenetic factors [ 10, 49]. Adipogenesis includes two stages: the commitment of MSCs to preadipocytes and the differentiation of preadipocytes to mature adipocytes [50]. PPARγ is the most important transcription factor in preadipocyte differentiation and is essential for lipid droplet deposition, insulin sensitivity, adipocyte survival, and function maintenance [9]. A-FABP plays an important role in the lipid metabolism of adipocytes [51]. Additionally, *A-FABP* is a marker gene of preadipocyte differentiation. The expression of A-FABP is increased significantly during the differentiation of preadipocytes [52]. FAS is a multifunctional enzyme that plays a central role in lipid biosynthesis and is responsible for the endogenous synthesis of fatty acids [53]. In addition, FAS plays an important role in preadipocyte differentiation [54]. In the current study, we used *ALKBH5*-knockout ICP2 cells as the cell model to investigate the function of ALKBH5 in chicken preadipocyte differentiation. The accumulation of lipid droplets and the expression of pro-adipogenesis genes ( *PPARγ*, *A-FABP*, and *FAS*) were significantly decreased in *ALKBH5*-knockout cells compared with those in the wild-type cells. These results demonstrated that ALKBH5 could promote chicken preadipocyte differentiation by directly or indirectly enhancing the expressions of *PPARγ*, *A-FABP*, and *FAS*, but further studies are required to understand the specific mechanism. A recent study reported that ALKBH5 could inhibit adipogenic differentiation of human MSCs [23]. Thus, we speculate that ALKBH5 may play different roles in different stages of adipogenesis.

In summary, in this study we demonstrated that the expression of ALKBH5 is associated with fat deposition at the early stage of chicken abdominal adipose tissue development. In addition, although further studies are needed to elucidate the molecular mechanism of ALKBH5-mediated preadipocyte proliferation and differentiation, our findings clearly identified ALKBH5 as a novel modulator of chicken preadipocyte proliferation and differentiation.

## Supporting information

294FigS1

## Supplementary Data

Supplementary data is available at *Acta Biochimica et Biphysica Sinica* online.
