# Supplementary figures and images for: N 6 -methyladenosine demethylase ALKBH5: a novel regulator of proliferation and differentiation of chicken preadipocytes : ALKBH5 regulated preadipocyte proliferation and differentiation
Source: Acta Biochim Biophys Sin (Shanghai). 2021 Dec 23;54(1):55–63. doi: 10.3724/abbs.2021007 (PMC10335918; doi:10.3724/abbs.2021007)

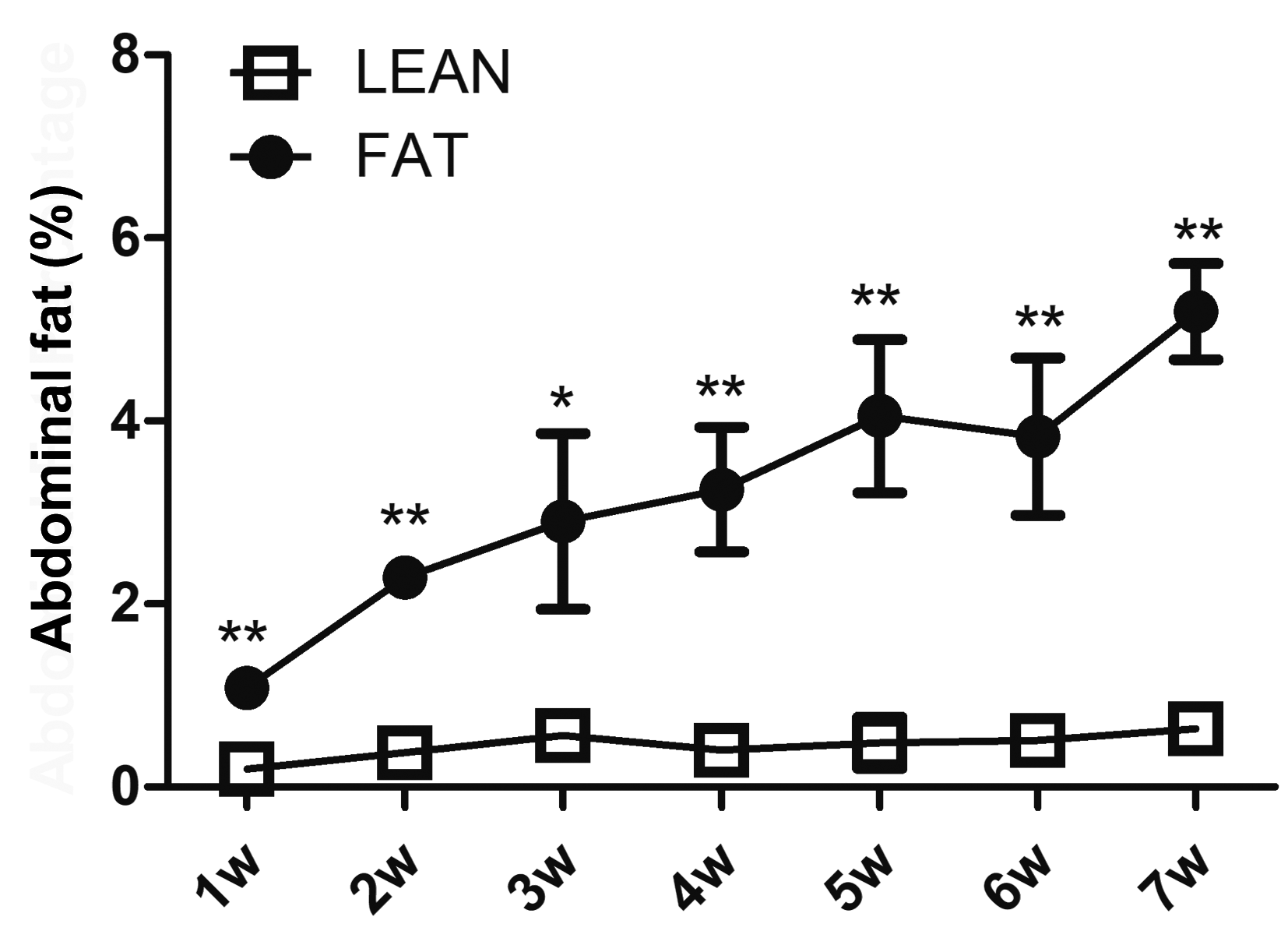

Supplement: 294FigS1 [file 294FigS1.tif]
